# Editorial: Unleashing Innovation on Precision Public Health–Highlights From the MCBIOS and MAQC 2021 Joint Conference

**DOI:** 10.3389/frai.2022.859700

**Published:** 2022-02-25

**Authors:** Ramin Homayouni, Huixiao Hong, Prashanti Manda, Bindu Nanduri, Inimary T. Toby

**Affiliations:** ^1^Oakland University William Beaumont School of Medicine, Rochester, MI, United States; ^2^National Center for Toxicological Research, United States Food and Drug Administration, Jefferson, AR, United States; ^3^University of North Carolina at Greensboro, Greensboro, NC, United States; ^4^College of Veterinary Medicine, Mississippi State University, Mississippi State, MS, United States; ^5^Department of Biology, University of Dallas, Irving, TX, United States

**Keywords:** machine learning, genomics, adverse drug effects, alternatives to animal testing, artificial intelligence

This Research Topic is a product of the 17th annual conference of the Midsouth Computational Biology and Bioinformatics Society (MCBIOS), which has a broad membership of scientists and trainees with research interests in genomics, medicine, drug discovery and therapeutics. The topic includes a total of eight papers, four of which appear in *Frontiers in Artificial Intelligence* (including three original research articles and one review), three in *Frontiers in Big Data* (including one original research article, one Technology and Code article, and one brief research report), and one in *Frontiers in Genetics Computational Genomics* (Data Report). The papers can be categorized into two general themes of genomics and machine learning applications, as described below.

## Genomics

Genomic data are generated in a complicated multi-step process that can impact the reproducibility of the results. In addition, many methods and software tools are available to analyze genomic data, which often yield different results from the same data. To address these challenges, Ma et al. developed a software infrastructure called NPARS (NGS post-pipeline accuracy and reproducibility system) that encapsulates genomic datasets in a portable database container, which can then be analyzed by well-established open-source application programming interfaces (APIs). They demonstrated the usefulness of NPARS in improving accuracy and reproducibility of different analysis methods on large and complex genomic data sets. In addition, the infrastructure provides a more convenient means to collaborate between groups.

Wang et al. enhanced the loci2path software for performing eQTL enrichment to identify enriched tissue specific pathways. The improved version includes additional pathways from PID, Reactome, and WikiPathways. The study uses over 13 million eQTLS from the Genotype Tissue Expression (GTEx) resource for 49 tissue types. Biological pathways that are likely to be involved in ten critical traits such as Alzheimer's disease, schizophrenia, and non-small cell lung cancer were identified. The software was shown to be valuable at uncovering new biological mechanisms of important traits.

Quintanillla et al. developed a comprehensive database for genes and variants specifically related to Acute Respiratory Distress Syndrome (ARDS). The ARDS-DB framework provides gene and variant information and associated metadata derived from primary level curation of experimentally verified studies. The advantage of a dedicated gene database for deeper analysis of ARDS is that it provides the user with a centralized location to retrieve pertinent information. ARDS DB is freely available *via* an open-source repository and represents a major step toward filling a gap in computational resources for bench biologists and clinicians.

## Machine Learning Applications

Scientific data are growing and expanding at an overwhelming pace, making it challenging for scientists to organize, analyze and extract value from the vast amount of data. There is an urgent need for efficient and reliable methods and tools to mine signatures out of large datasets. Using an unsupervised machine learning approach, Nguyen et al. developed a software tool called SEAS (Statistical Enrichment Analysis of Samples) for mining biological sample information from genomic data. SEAS is available as a standalone or web version with a user-friendly graphical user interface. It can extract metadata and analyze numerical and categorical data to compute sample similarities and to cluster samples (e.g., patients). The authors demonstrated the utility of SEAS on publicly available data sets from The Cancer Genome Atlas (TCGA).

Li et al. present the development and implementation of DeepCarc, which uses a deep learning framework to predict carcinogenicity of small molecules. DeepCarc was developed using data in the National Center for Toxicological Research liver cancer database (NCTRlcdb) and tested against data in DrugBank and Tox21. DeepCarc model outperformed five machine learning classifiers, two state-of-the-art ensemble methods, and four molecule-based deep learning models. The DeepCarc model is designed to be an alternative method to test carcinogenicity and to alleviate the time-consuming and labor-intensive process of evaluating carcinogenic potency in experimental animal systems. DeepCarc is freely available for use and can be accessed *via* the following link: (https://github.com/TingLi2016/DeepCarc).

Application of machine learning to histopathological images is becoming common in both academic and commercial domains. There is still a need to detect and classify different immune cell types in the tumor immune microenvironment (TIME), which play crucial roles in determining cancer progression, metastasis, and response to treatment. Lee et al. provide a review of published models and applications in the three different scales of histopathology analyses: whole slide image (WSI)-level, region of interest (ROI)-level, and cell-level. In addition they provide a simplified framework for the development of a cell-type classifier using weakly labeled datasets generated from immunolabeled slides. The pros and cons for each method is highlighted and the future direction for histopathological image analysis is discussed.

Automated analysis of drug labels for “indication and usage” can be useful for clinical decision making, regulatory management as well as drug repositioning. Bhatt et al. developed a five-category Drug Indication Classification and Encyclopedia (DICE) based on >7,000 sentences from FDA approved human prescription drug labels. In addition, they developed nine different AI-based classifiers, including 4-word embeddings-based Bidirectional long short-term memory (BiLSTM) models and five transformer-based language models. The model performance was comprehensively assessed based on a test set and an independent validation set.

Adverse drug reactions (ADRs) such as drug-induced liver injury (DILI) are described in three sections, “Adverse Reactions”, “Warnings and Precautions” and “Boxed Warning”, in FDA drug labeling documents. Because of the complexity of the language and lack of standardization, Wu et al. explored using deep learning based language modeling approach to classify DILI from drug labels. A Bidirectional Encoder Representations from Transformers (BERT) model was trained for binary DILI classification of FDA-approved drug labeling documents and was externally validated using EMA-approved drug labeling documents.

Taken together, the papers selected for this Research Topic provide examples of cutting-edge approaches for standardizing analysis of large datasets and demonstrate the utility of applying machine learning methods to extract valuable insights from such data sources.

## Author Contributions

All authors listed have made a substantial, direct, and intellectual contribution to the work and approved it for publication.

## Funding

PM is supported by a grant from the Division of Biological Infrastructure at the National Science Foundation (# 1942727). BN is partially supported by grant # P20GM103646 (Center for Biomedical Research Excellence in Pathogen Host Interactions) from the National Institute for General Medical Sciences. IT is supported by a grant from The Nancy Cain and Jeffrey A. Marcus Science Endowment in Honor of President Donald A. Cowan (University of Dallas).

## Author Disclaimer

This editorial reflects the views of the authors and does not necessarily reflect those of the U.S. Food and Drug Administration.

## Conflict of Interest

The authors declare that the research was conducted in the absence of any commercial or financial relationships that could be construed as a potential conflict of interest.

## Publisher's Note

All claims expressed in this article are solely those of the authors and do not necessarily represent those of their affiliated organizations, or those of the publisher, the editors and the reviewers. Any product that may be evaluated in this article, or claim that may be made by its manufacturer, is not guaranteed or endorsed by the publisher.

